# Emotional competences as predictors of psychological wellbeing and quality of life of supplementary grandparents caregivers

**DOI:** 10.3389/fpsyg.2024.1411634

**Published:** 2024-08-27

**Authors:** Leyre Galarraga, Cristina Noriega, Gema Pérez-Rojo, Javier López

**Affiliations:** ^1^Department of Psychology and Psychopedagogy, Faculty of Medicine, CEU San Pablo University, Madrid, Spain; ^2^Grupo de Investigación Envejecimiento (BUENA VEJEZ), Madrid, Spain; ^3^Instituto de Estudios de las Adicciones (IEA-CEU), Madrid, Spain

**Keywords:** grandparents caregivers, grandparenthood, caregiving, psychological wellbeing, quality of life, emotional competences, role meaning, caregiving overload

## Abstract

**Introduction:**

Grandparents are increasingly becoming key figures in the supplementary care of grandchildren. Based on the Resilience Model of Family Stress, Adjustment and Adaptation, the present study aims to analyze the emotional competences that canpl predict higher levels of psychological wellbeing and quality of life in supplementary grandparents caregivers.

**Methods:**

A sample of 270 supplementary grandparents caregivers living in Spain participated. Most participants were women (71.1%), and the mean age was 67.83 years (SD = 6.26). Most participants were occasional caregivers, that is, they care for less than 10 h per week (76.3%). We conducted hierarchical multiple regression analysis, one for psychological wellbeing and the other for quality of life.

**Results:**

The regression model for psychological wellbeing identified that age, management of caregiving stress, self-confidence in the caregiving role, management of work-life balance difficulties and emotional self-regulation explained 32.8% of its variance. The regression model for quality of life showed that age, type of grandparent caregiver, management of caregiving stress, management of work-life balance difficulties and emotional self-regulation explained 31.2% of its variance.

**Conclusion:**

This study focuses on supplementary grandparents caregivers, whereas literature has tended to look at primary grandparents caregivers. The results highlight the role of emotional competences as predictors of supplementary grandparents caregivers’ psychological wellbeing and quality of life, overcoming the usual tendency in the literature to focus on the negative consequences of grandparents caregiving for grandchildren, and emphasizing the competences that grandparents have to cope with this care in a satisfactory way, which, moreover, can be trained.

## 1 Introduction

Western societies have undergone sociodemographic changes that have favored the development of grandparents’ caregiving role, such as increased life expectancy (Chapman et al., 2017), delay of first birth, decline in fertility (Cisotto et al., 2022), changes in the labor world (Di Gessa et al., 2016), apparition of new family types (Di Gessa et al., 2016), and an increase in the social participation of the older adults (Cisotto et al., 2022). These sociodemographic changes have caused changes in family relationships, becoming grandparents key figures in helping younger generations to reconcile family and work life. This intergenerational help is transmitted, fundamentally, by providing supplementary care to grandchildren (Geurts et al., 2015).

The degree of involvement of grandparents as caregivers of their grandchildren can be understood as a continuum. At one pole, there are the primary grandparents caregivers, those who provide permanent care due to the temporary or total absence of the parents. At the other pole, there are supplementary grandparents caregivers, those who provide care in addition to the parental care, as a way of supporting grandchildren’s parents (Villar et al., 2012).

Taking care of a grandchild occasionally (as a voluntary choice and leisure activity) may not be as demanding as taking on the responsibility of caregiving regularly. For this reason, literature differentiated between occasional (less than 10 h of care per week) and regular supplementary caregivers (10 h of care or more per week) (Wellard, 2011; Noriega et al., 2022).

Zanasi et al. (2023) analyzed the proportion and intensity of supplementary care provided by grandparents in Europe, and determined that 46% of participants had provided *occasional care* to their grandchildren between 2019 and 2020. The lowest proportion was in Latvia (24%) and the highest in Belgium and Netherlands (60%). Mediterranean countries occupied the middle positions (Spain, 43%; Greece, 38%; and Italy, 37%). Analyzing *regular care*, they determined that the proportion of grandparents providing it was 25% between 2019 and 2020. The lowest proportion was in Latvia (12%) and the highest in Belgium (39%). Mediterranean countries occupied the middle positions (Italy, 31%; Spain 25%; and Greece, 25%).

The most recent data provided by the Survey on Health, Aging and Retirement in Europe (SHARE; Börsch-Supan, 2022), showed that 60% of grandparents provided supplementary care to their grandchildren. Specifically, 30% of grandparents cared for their grandchildren at least 30 h per month. Considering those who cared regularly (daily care), the Spanish percentage was 12%.

Although the above figures reflect the high percentage of supplementary grandparent caregivers, the literature has focused on primary grandparents caregivers (Villar et al., 2012; Triadó et al., 2014). The Resilience Model of Family Stress, Adjustment and Adaptation (McCubbin, 1993) explains why some families are more resilient than others, having a greater capacity to adapt to family stress. The model analyses the family as a single unit, focusing on three aspects that influence its adjustment and adaptation process: (1) Family demands, the stimuli that pressure the family system to adjust and adapt; (2) family resources, which include those of each family member, those of the family in general, those of the community, and those that arise in response to the family demands; and (3) problem-solving skills, which refer to the strengths that the family has and how it uses them to adjust and adapt to family demand.

**Figure 1:**
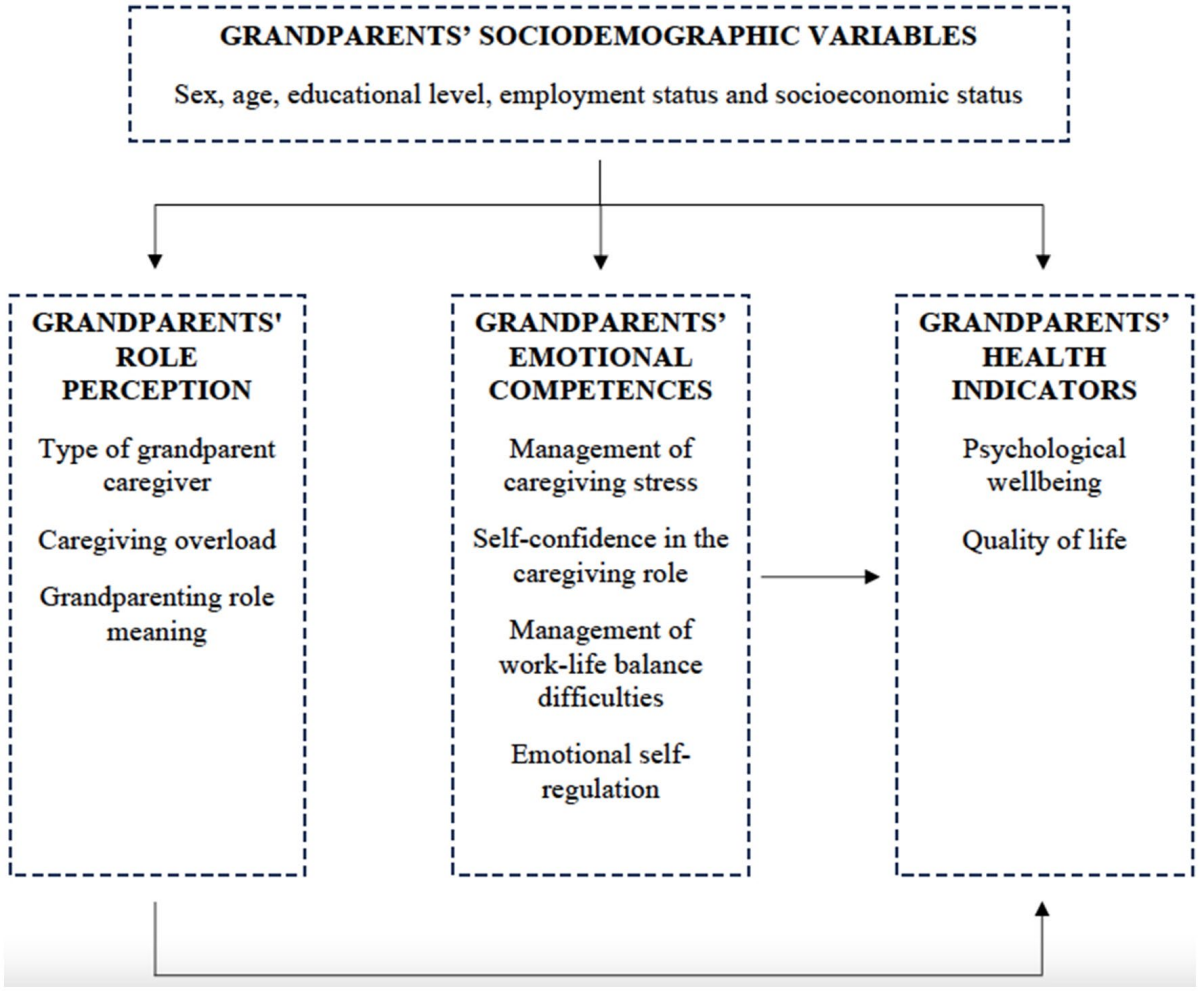
Application of the Resilience Model of Family Stress, Adjustments and Adaption to the present study.

Building on research in which this model was used in the study of family coping with health crises, Musil et al. (2006, 2009) applied it to understand the experience of primary grandparents caregivers. Their main hypothesis was that, if family demands were not buffered by grandparents’ resources, their health could be affected. Subsequently, Noriega et al. (2022) applied this model to grandparents who provide supplementary care in Spain. The authors first studied the impact of the amount of care provided on grandparents’ health-related QoL, and then, the protective role of social support and character strengths, finding that both variables mediated the impact of the amount of care provided on grandparents’ health-related quality of life (QoL). Figure 1 shows the application of this model to the present research.

Although there is consensus on the lower psychological impact suffered by grandparents providers of supplementary care versus primary caregivers (Glaser et al., 2014), there are different levels of psychological impact within the group of supplementary caregivers, depending on the intensity of care. The psychological impact of supplementary grandchild care on grandparents has been commonly assessed with two indicators: wellbeing (Villar et al., 2012; Danielsbacka et al., 2019) and QoL (Szabó et al., 2019; Hemmati et al., 2023).

Concerning *wellbeing*, most studies have analyzed subjective wellbeing (SW), using indicators such as happiness, positive and negative affect, and life satisfaction. Most of these studies agree that moderate levels of caregiving are related to more life satisfaction (Moore and Rosenthal, 2015) and feelings of usefulness (Danielsbacka et al., 2019), as well as lower depressive symptoms (Xu et al., 2017), anxiety and stress, and feelings of loneliness (Tang et al., 2016). Other studies have evidenced that intense levels of caregiving increase grandparents’ risk of depression (Brunello and Rocco, 2016; Komonpaisarn and Loichinger, 2019).

In contrast, the number of studies considering psychological wellbeing (PW) is scarce. It refers to people’s feelings and behaviors aimed at developing their potential and giving meaning to their lives. According to Ryff’s model (1989), PW is composed of six dimensions: self-acceptance (positive attitude toward oneself, accepting qualities and limitations), vital purpose (feeling of direction in life, toward the past and the future), mastery of the environment (competence in managing situations and opportunities), positive relationships (building satisfying and trusting bonds), personal growth (feeling of continuous development, having made use of one’s talents), and autonomy (independence of thought and behavior, with congruence of conduct-personal convictions). This model has been used to study the relationship between Erikson’s (2000) life cycle stages and PW, to analyze the transitions that take place in adulthood (e.g., retirement), as well as to understand the impact of significant life events (e.g., caring for a child with a disability) (Ryff, 2014). Compared to SW, it is more stable over time, so it seems more relevant to know the impact of grandchild care on PW.

Regarding *QoL*, the World Health Organization (1995) defines it as the expression of how people perceive their attitudes toward life, considering their cultural and value context, concerning their goals, expectations, interests, standards, concerns, and lifestyle. Higgs et al. (2003) and Hyde et al. (2003) developed a model of QoL based on the satisfaction of human needs, based on Maslow (1943). They identified four human needs that would constitute the dimensions of QoL: autonomy (right of people to be independent of the influence of others), control (ability to manage one’s environment), pleasure (development of activities that provide agreeable emotions), and self-fulfillment (using one’s capabilities to develop personal identity).

Researchers found higher scores on psychological and physical dimensions of QoL in grandparents providers of supplementary care, versus non-caregivers or non-grandparents (Tanskanen et al., 2019; Hemmati et al., 2023). Di Gessa et al. (2023) evidenced lower QoL in grandparents who completely stopped caring for their grandchildren during the COVID-19 pandemic, while those who maintained or even increased caregiving increased their QoL. In contrast, Szabó et al. (2019), studying not only supplementary grandparent caregivers, but also older people who cared for children outside their families, found different relationships of caregiving with dimensions of psychosocial quality of life. Caregiving was negatively related to the control and autonomy dimension in men, while caregiving was positively related to the self-fulfillment dimension in women.

With respect to grandparents’ role perception, previous researches in this field have suggested that is not only important the amount of care provided, but also the perception of this care as a source of discomfort or satisfaction, which may also influence their health (González et al., 2015; Xu et al., 2017). In this sense, type of grandparent caregiver, caregiving overload and grandparental role meaning have been related to grandparents’ wellbeing (Hayslip et al., 2003; Triadó et al., 2014).

Related to *type of grandparent caregiver* and *caregiving overload*, intensity of caregiving is one of the caregiving variables that has received the most attention in the literature because of its important role in the impact that caregiving can have on grandparents’ health. Literature agrees that caregiving has health benefits for grandparents when it is not intense, whereas it can be detrimental when the intensity is excessively high (Glaser et al., 2014; Burn and Szoeke, 2015). Triadó et al. (2014) evidenced that more hours of care were associated with more perceived difficulties by grandparents, and that these difficulties were negatively associated with role satisfaction. The predictors of the subjective health of grandparents found were age, amount of care provided and perceived difficulties, the latter being the most significant. Consequently, the authors concluded that it is not so much the amount of care provided, but the perception of that care (Triadó et al., 2014). Tang et al. (2016) and Xu et al. (2017) observed a positive relationship between caregiving overload and caregiving pressure (pressure from parents to grandparents to take care of grandchildren) with different indicators of subjective wellbeing (depressive symptoms, anxiety, stress and loneliness). Brunello and Rocco (2016) likewise evidenced an increased risk of depression in grandparents when caregiving for their grandchildren was excessively intense. Regarding PW, studies are scarce, StGeorge and Fletcher (2014) and Villar et al. (2012) reported that involvement in the care of grandchildren favored the understanding of the past, the construction of future purposes, as well as the feeling of being a guide for the new generation, which enhanced the PW of grandparents. About QoL, known studies have compared supplementary grandparents caregivers and non-caregivers grandparents, showing a higher QoL in the first one. However, there are no known studies that have compared QoL between occasional and regular supplementary grandparent caregivers.

In relation to the *grandparental role meaning*, supplementary caregivers have reported the following meanings of their grandparenthood: love, pleasure, energy, joy, peace, gratitude for being able to care a second time, opportunity to strengthen family ties, contribution to the family, and meaning and purpose in life (Villar et al., 2012; Triadó et al., 2014; Fauziningtyas et al., 2018; Şahin and Şahin, 2020). Thiele and Whelan (2008) found significant positive relationships between four of Kivnick’s (1982, 1983) grandparental meanings (valued older, centrality, immortality, and reinvolvement) and role satisfaction. However, valued older and centrality meanings, with generativity, were the main predictors of role satisfaction. Although there are no known studies that have evaluated the relationship between grandparental role meaning and QoL, the literature shows that the more grandparents identify with their role meaning, the greater their wellbeing, their satisfaction with their grandparental role and the quality of their relationship with their grandchildren (Kivnick, 1982, 1983; Hayslip et al., 2003).

Last of all, *emotional competences* can be defined as the capacities, knowledge, skills, and attitudes necessary to understand, express and regulate emotions (Bisquerra and Pérez, 2007). Emotional intelligence is a well-known psychological strength, related to resilience, that favors adaptation in stressful circumstances, thanks to emotional self-awareness, expression, and management (Armstrong et al., 2011). Starting from the existing literature on grandparents supplementary caregivers, as well as from parental competency assessment instruments, García and Martínez-González (2017) proposed that supplementary grandparents caregivers must have the necessary emotional competences to attend to their growing responsibilities in current society. This is the only research, to our knowledge, describing the emotional competences of grandparents, in which four emotional competences were described: management of caregiving stress, self-confidence in the caregiving role, management of work-life balance difficulties, and emotional self-regulation (García and Martínez-González, 2017).

Despite the fact that there are no known studies analyzing the relationship between grandparents’ emotional competences, type of grandparent caregiver, caregiving overload, grandparental role meaning, PW and QoL, there are some intervention studies that shed light on this question. Some authors have developed intervention programs focused on teaching supplementary grandparent caregivers’ different psychological resources, which resemble emotional competences, to improve their health.

Kirby and Sanders (2014) and Leung and Fung (2014) taught grandparents parenting strategies (updating education and parenting tools), team strategies (building pleasant relationships with parents) and coping strategies (emotional management). Zauszniewski et al. (2014) taught grandparents eight cognitive-behavioral tools for coping with adversity, three resources for seeking help from others, and five self-help resources. Strom and Strom (2016) offered their participants the possibility to share experiences about grandparenting and to have tools to become aware of how children’s education had changed. On the one hand, these interventions achieved a reduction in symptoms of depression, anxiety and stress (Kirby and Sanders, 2014; Leung and Fung, 2014; Zauszniewski et al., 2014), and an increase in grandparents’ health-related QoL (Zauszniewski et al., 2014). On the other hand, authors observed an increase in role satisfaction and role meaning (Strom and Strom, 2016) and in self-efficacy (Kirby and Sanders, 2014; Leung and Fung, 2014; Strom and Strom, 2016). Parental self-efficacy refers to parents’ confidence in their ability to overcome parenting challenges and promote their children’s development (Coleman and Karraker, 2000), and has been negatively associated with parental stress (Ayala-Nunes et al., 2014).

Based on the Resiliency Model of Family Stress, Adjustment and Adaptation of McCubbin (1993), this study addresses the following research questions (1) Are there significant differences in PW and QoL by grandparents’ sociodemographic variables?; (2) Are there significant relationships between PW and QoL and grandparents’ emotional competences and grandparents’ role perception?; (3) Do emotional competences, amount of care provided, role meaning and caregiving overload predict PW?; and (4) Do emotional competences, amount of care provided, role meaning and caregiving overload predict QoL? Considering these research questions, we hypothesized that (1) the older the grandparents’ age, the lower the PW and QoL; (2) PW and QoL will be positively related to the four *emotional competences* and role meaning, and negatively to caregiving overload, and there will be no difference depending on the type of caregiver; (3) higher scores on the four emotional competences, being an occasional grandparent caregiver, higher score on role meaning and lower score on caregiving overload will predict positively PW; and (4) higher scores on the four emotional competences, being an occasional grandparent caregiver, higher score on role meaning and lower score on caregiving overload will predict positively QoL.

## 2 Methods

### 2.1 Participants

The inclusion criterion was to be a supplementary grandparent caregiver of, at least, one grandchild under 18. In other words, any grandparent who cared for their grandchildren in an auxiliary way to the parents’ upbringing, as a support figure for the reconciliation of family and work life, could participate in the study. To identify grandparents who were supplementary caregivers, the following item was included: *“Have you ever care for your grandchild(ren) in the last 12 months?.”* Consequently, the exclusion criteria were both having a pathology that impeded participation in the study and being a primary grandparent caregiver, that is, being the main caregiver of the grandchildren in the absence of the parents.

Participants were 270 supplementary grandparents caregivers living in Spain. Most of the participants were women (71.1%). The mean age was 67.83 years (*SD* = 6.26), with an age range from 51 to 88 years. Most of the participants were married or cohabiting with their partners (65.9%), had a medium socioeconomic level (58.1%), were retired (73.3%) and had university studies (58.5%).

Most participants were occasional caregivers (76.3%) and cared for their grandchildren weekly (41%). The mean number of grandchildren cared for in the last 12 months was 2.22 (*SD* = 1.64). The mean age of the grandchildren was 5.73 (*SD* = 3.83), with an age range from newborn to 18 years. Most participants cared for grandsons and granddaughters (41.1%) and mainly cared for their daughters’ children (64.1%) (Table 1).

**Table 1:** Sociodemographic characteristics of grandparents and characteristics of caregiving.

Category	Total sample (*N* = 270)
	*Mean (SD)*/*N* *(%)*
Grandparents’ age	67.83 (6.26)
Grandparents’ sex	
Woman	192 (71.1%)
Man	78 (28.9%)
Grandparents’ marital status	
Married or cohabiting with a partner	178 (65.9%)
Divorced or separated	47 (17.4%)
Widow/widower	45 (16.7%)
Place of residence	
Community of Madrid	115 (42.6%)
Castilla y León	22 (8.1%)
Castilla-La Mancha	3 (1.1%)
Principado de Asturias	2 (0.7%)
Cataluña	27 (10%)
Extremadura	2 (0.7%)
Región de Murcia	4 (1.5%)
Comunidad Valenciana	71 (26.3%)
País Vasco	11 (4.1%)
Galicia	5 (1.9%)
Comunidad Foral de Navarra	1 (0.4%)
Islas Baleares	2 (0.7%)
Aragón	1 (0.4%)
Andalucía	4 (1.5%)
Grandparents’ socioeconomic status	
Low	5 (1.9%)
Medium-low	35 (13%)
Medium	157 (58.1%)
Medium-high	70 (25.9%)
High	3 (1.1%)
Grandparents’ employment status	
Employee	27 (10%)
Self-employed	16 (5.9%)
Unemployed	11 (4.1%)
Home care	16 (5.9%)
Retired	198 (73.3%)
Disability	1 (0.4%)
Student	1 (0.4%)
Grandparents’ educational level	
No education	1 (0.4%)
Primary education	33 (12.2%)
Secondary education	78 (28.9%)
University education	158 (58.5%)
Type of caregiver	
Occasional	206 (76.3%)
Regular	64 (23.7%)
Frequency of care	
Daily	48 (17.8%)
Weekly	111 (41%)
Monthly	59 (21.9%)
With less frequency	52 (19.3%)
Number of grandchildren cared for	2.22 (1.64)
Grandchildren age	5.73 (3.83)
Grandchildren sex	
Grandson	72 (26.7%)
Granddaughter	87 (32.2%)
Grandson and granddaughter	111 (41.1%)
Family linage	
Paternal	173 (64.1%)
Maternal	56 (20.7%)
Both	41 (15.2%)

### 2.2 Variables and instruments

#### 2.2.1 Sociodemographic data of grandparents

The following variables were included: sex, age, marital status, socioeconomic status, employment status, and educational level.

#### 2.2.2 Intergenerational data

We analyzed the type of caregiver (occasional caregiver, if they were caring for their grandchildren less than 10 h per week; or regular caregiver, providers of care for 10 or more hours per week), number of grandchildren cared for in the last 12 months, grandchildren’s age, grandchildren’s sex, and family lineage (maternal, paternal, or both).

The type of grandparent caregiver (occasional or regular) has been taken into account in order to control the influence of the intensity of care, since the literature shows that, even if the care is auxiliary, if it is excessively intense, it can affect the grandparent’s health.

#### 2.2.3 Psychological wellbeing

We used the Ryff scale of psychological wellbeing (1989; Spanish brief version validated by Díaz et al., 2006). This instrument includes 29 items organized in 6 dimensions: self-acceptance, positive relationships, autonomy, mastery of the environment, personal growth, and purpose in life. Items are scored from 1 “strongly disagree” to 6 “strongly agree.” An example of an item from the self-acceptance dimension is: *“For the most part, I am proud of who I am and the life I lead”*; from the positive relationships dimension is: *“I know I can trust my friends, and they know they can trust me”*; from the autonomy dimension is: *“I am concerned about how other people evaluate the choices I have made in my life”*; from the mastery of the environment dimension is: *“I have been able to build a home and a way of life to my liking”*; from the personal growth dimension is: *“Overall, over time I feel like I continue to learn about myself”*; and from the purpose in life dimension is: *“I am an active person in carrying out the projects I proposed for myself.”* We used the total score, which showed good reliability (*Cronbach’s α* = 0.89).

#### 2.2.4 Quality of life

It was applied the QoL questionnaire for older adults (CASP-12; Spanish validation by Pérez-Rojo et al., 2017). This scale was used to study the quality of life through 12 items organized in three dimensions: control and autonomy, pleasure, and self-fulfillment. Items are scored from 1 “often” to 4 “never.” An example of an item from the control and autonomy dimension is: *“My age prevents me from doing the things I would like to do”*; from the pleasure dimension is: *“I look forward to every day”*; and from the self-fulfillment dimension is: *“I believe my future looks bright.”* We used the total score which showed good reliability (*Cronbach’s α* = 0.87).

#### 2.2.5 Emotional competences

We used the Grandparents’ emotional competences scale (ECEA; García and Martínez-González, 2017). This instrument includes 10 items distributed in 4 dimensions: management of caregiving stress, self-confidence in the caregiving role, management of work-life balance difficulties and emotional self-regulation. Items are scored from 1”never” to 4 “always.” An example of an item from the management of caregiving stress dimension is: *“With my grandchild, I have the feeling that I have not achieved everything I had hoped for”*; from the self-confidence in the caregiving role dimension is: *“I believe that I am a good grandparent and that I fulfill my duties adequately”*; from the management of work-life balance difficulties dimension is: *“I feel overwhelmed by the circumstances, with no time to take care of my grandchild”*; and from the emotional self-regulation dimension is *“I know how to relax and control my emotions in front of my.”* In our sample, all the subscales showed acceptable reliability (*Cronbach’s α* = 0.63–0.76).

#### 2.2.6 Grandparenting role meaning

We used the Multidimensional experience of grandparenthood set of inventories (Findler et al., 2013). We applied the dimension of role meaning, which consists of 8 items that range from 1 “completely disagree” to 5 “completely agree.” An example of an item is: *“Being a grandparent gives more meaning to my life.”* In our sample, this instrument showed acceptable reliability (*Cronbach’s α* = 0.78).

#### 2.2.7 Caregiving overload

We used the Multidimensional experience of grandparenthood set of inventories (Findler et al., 2013). We applied the dimension of caregiving overload, which consists of 4 items ranging from 1 “completely disagree” to 5 “completely agree.” An example of an item is: “For me, being a grandparent is a real burden.” In our sample, this instrument showed acceptable reliability (*Cronbach’s α* = 0.74).

### 2.3 Procedure

First, the project was approved by San Pablo CEU University Ethics Committee (516/21/40). The data for this study were collected between March 2021 and June 2023. The sampling was a convenience sampling. The sample was collected from different organizations, social centers, universities for seniors, and using the snowball sampling technique. Then, participants completed a self-administered questionnaire (in electronic or paper format), which took between 20 and 30 min. Informed consent was obtained from all respondents and the confidentiality of their data was explicitly guaranteed.

### 2.4 Data analysis

The IBM SPSS version 27 statistical package was used for data analysis.

Firstly, a univariate analysis was carried out to study the differences in PW and QoL as a function of the sociodemographic variables of the grandparents. Mann–Whitney U tests was used with the variable sex, Spearman correlations with age, and Kruskal Wallis tests with marital status, socioeconomic status, employment status and educational level. After performing the latter tests, in those where significant differences were observed, Mann–Whitney U tests was performed to see between which levels of the variables the differences were found.

Secondly, the relationships of the dependent variables (PW and QoL) with the grandparents’ emotional competences and grandparents’ role perception variables were studied using Mann–Whitney U tests (for the variable type of caregiver) and Spearman correlations (for the rest of the variables).

Thirdly, the variables showing statistically significant relationships with PW and QoL were included in Hierarchical multiple regression analysis.

## 3 Results

Based on the Resilience Model of Family Stress, Adjustment and Adaptation (McCubbin, 1993) we studied the relationships between grandparents’ sociodemographic variables, grandparents’ role perception and grandparents’ emotional competences with two health indicators (PW and QoL).

### 3.1 Relationship of psychological wellbeing and quality of life with grandparents’ sociodemographic variables and intergenerational data

Concerning PW, a statistically significant relationship was found with *age* (*r* = −0.24, *p* < 0.001). Regarding *sex*, statistically significant differences were found, with the mean score being higher for women. Statistically significant differences were found in *employment status* (Table 2). Mann–Whitney tests were performed to analyses between which levels of the employment status variable the differences were found. First, statistically significant differences (*U* = 1,457; *p* < 0.001) were found between employee (*M* = 158.04) and retired (*M* = 106.86). Second, statistically significant differences (*U* = 1,042.5; *p* < 0.005) were found between self-employed (*M* = 141.34) and retired (*M* = 104.77). No statistically significant differences were found according to *marital status*, *socioeconomic status,* and *educational level*, *number of grandchildren cared*, and *grandchildren’s age*.

**Table 2:** Differences in psychological wellbeing according to sex and employment status.

		*Median*	*U*/*H*	*p*-value
Sex	Women	141.89	6,262	0.035
	Men	119.78		
Employment status	Employee	185.96	20.603	0.002
	Self-employed	169.47		
	Unemployed	155.09		
	Home care	141.13		
	Retired	123.83		
	Disability	215		
	Student	154.5		

About QoL, a statistically significant relationship was found with *age* (*r* = −0.24, *p* < 0.001). Statistically significant differences were found in *socioeconomic status, employment status*, and *educational level* (Table 3). Mann–Whitney tests were performed to analyses between which levels of the variables the differences were found.

**Table 3:** Differences in quality of life according to socioeconomic status, employment status and educational level.

		*Median*	*H*	*p*-value
Socioeconomic status	Low	98.2	12.428	0.014
	Medium-low	110.66		
	Medium	131.87		
	Medium-high	160.03		
	High	105		
Employment status	Employee	171.59	15.834	0.015
	Self-employed	171.81		
	Unemployed	144.14		
	Home care	123.19		
	Retired	127.08		
	Disability	242		
	Student	242		
Educational level	No education	25.5	8.098	0.044
	Primary education	105.55		
	Secondary education	135.62		
	University education	142.39		

Regarding *socioeconomic status*, first, statistically significant differences (*U* = 780; *p* < 0.005) were found between medium-low (*M* = 40.29) and medium-high (*M* = 59.36). Second, statistically significant differences (*U* = 4,356.5; *p* < 0.005) were found between medium (*M* = 106.5) and medium-high (*M* = 130.26).

About *employment status*, first, statistically significant differences (*U* = 132; *p* < 0.005) were found between employee (*M* = 25.11) and home care (*M* = 16.75). Second, statistically significant differences (*U* = 1793; *p* ≤ 0.005) were found between employee (*M* = 145.57) and retired (*M* = 108.56). Third, statistically significant differences (*U* = 1,050; *p* ≤ 0.005) were found between self-employed (*M* = 140.88) and retired (*M* = 104.8).

In relation to *educational level*, statistically significant differences (*U* = 1871; *p* < 0.005) were found between primary education (*M* = 73.7) and university education (*M* = 100.66).

No statistically significant differences were found according to *sex* and *marital status*.

### 3.2 Relationship of psychological wellbeing and quality of life with grandparents’ role perception and grandparents’ emotional competences

The correlations between *PW*, *QoL*, *grandparents’ role perception*, and *grandparents’ emotional competences* are shown in Table 4.

**Table 4:** Correlations between psychological wellbeing, quality of life, grandparents’ emotional competences and grandparents’ role perception.

	1	2	3	4	5	6	7	8
1. Psychological wellbeing	–	0.6^***^	0.38^***^	0.3^***^	0.37^***^	0.39^***^	0.228^***^	−0.311^***^
2. Quality of life		–	0.36^***^	0.28^***^	0.4^***^	0.34^***^	0.235^***^	−0.382^***^
3. Management of stress			–	0.27^***^	0.45^***^	0.26^***^	0.183^**^	−0.363^***^
4. Self-confidence				–	0.22^***^	0.33^***^	0.301^***^	−0.158^**^
5. Work-life balance					–	0.28^***^	0.255^****^	−0.502^***^
6. Emotional regulation						–	0.23^**^	−0.275^***^
7. Role meaning							–	−0.242^***^
8. Caregiving overload								–

^***^p ≤ 0.001; ^**^p ≤ 0.01; ^*^p ≤ 0.05.

Regarding the *type of grandparent caregiver*, statistically significant differences were only found in QoL (*U* = 5,187; *p* ≤ 0.01), with the mean score being higher for occasional caregivers (*M* = 141.89).

### 3.3 Role of statistically significant variables in psychological wellbeing

Hierarchical multiple linear regression was used to explain the influence of all variables previously described as statistically significant on grandparents’ PW. Following the Resilience Model of Family Stress, Adjustment and Adaptation (McCubbin, 1993) we included three blocks: (1) *sociodemographic variables* (age and sex); (2) *grandparents’ role perception* (grandparenting role meaning and caregiving overload); and (3) *emotional competences* (management of caregiving stress, self-confidence in the caregiving role, management of work-life balance difficulties and emotional self-regulation).

The final model (Table 5) identified five predictors of PW (age, management of caregiving stress, self-confidence in the caregiving role, management of work-life balance difficulties, and emotional self-regulation), which explain 32.8% of the variance of PW (*R*^2^ = 0.328; *adjusted R*^2^ = 0.308; *F*_(8,261)_ = 15.954).

**Table 5:** Hierarchical multiple linear regression model of psychological wellbeing.

	Parameter	*b*	*Standard error*	*t*	*p*-value	*β*	*R*^2^ *change*
Block 1	Intersection	179.855	12.205	14.737	<0.001		0.066^**^
	Age	−0.660	0.171	−3.864	<0.001	−0.238	
	Sex	2.021	2.362	0.856	0.393	0.053	
Block 2	Intersection	161.501	14.057	11.489	<0.001		0.093^**^
	Age	−0.554	0.164	−3.378	<0.001	−0.199	
	Role meaning	0.570	0.212	2.686	0.008	0.161	
	Caregiving overload	−1.704	0.446	−3.821	<0.001	−0.227	
Block 3	Intersection	64.010	18.006	3.555	<0.001		0.169^**^
	Age	−0.429	0.152	−2.812	0.005	−0.154	
	Role meaning	0.287	0.196	1.465	0.144	0.081	
	Caregiving overload	−0.034	0.490	−0.069	0.945	−0.005	
	Stress management	1.793	0.587	3.055	0.002	0.187	
	Self-confidence	1.915	0.760	2.520	0.012	0.138	
	Work-life balance	1.771	0.606	2.923	0.004	0.199	
	Emotional regulation	3.295	0.835	3.946	<0.001	0.223	

^**^p < 0.001.

On the one hand, management of caregiving stress, self-confidence in the caregiving role, management of work-life balance difficulties and emotional self-regulation explained PW with a positive relationship, that is, higher scores on these variables predicted higher scores on PW. On the other hand, age explained PW with a negative relationship, that is, higher score on this variable predicted a lower score on PW.

### 3.4 Role of statistically significant variables in quality of life

Hierarchical multiple linear regression was used to explain the influence of all variables previously described as statistically significant on grandparents’ QoL. Following the Resilience Model of Family Stress, Adjustment and Adaptation (McCubbin, 1993), we included three blocks: (1) *sociodemographic variables* (age); (2) *variables related to the grandparents’ role perception* (type of grandparent caregiver, grandparenting role meaning and caregiving overload); and (3) *emotional competences* (management of caregiving stress, self-confidence in the caregiving role, management of work-life balance difficulties and emotional self-regulation).

The final model (Table 6) identified five predictors (age, type of grandparent caregiver, management of caregiving stress, management of work-life balance difficulties, and emotional self-regulation), which explain 31.2% of the variance of QoL (*R*^2^ = 0.312; *adjusted R*^2^ = 0.291; *F*_(8,261)_ = 14.801).

**Table 6:** Hierarchical multiple linear regression model of quality of life.

	Parameter	*b*	*Standard error*	*t*	*p*-value	*β*	*R*^2^ *change*
Block 1	Intersection	55.471	4.281	12.959	<0.001		0.054^**^
	Age	−0.246	0.063	−3.923	<0.001	−0.233	
Block 2	Intersection	44.260	5.359	8.259	<0.001		0.145^**^
	Age	−0.199	0.059	−3.367	<0.001	−0.188	
	Type of caregiver	2.716	0.871	3.118	0.002	0.174	
	Role meaning	0.186	0.078	2.375	0.018	0.138	
	Caregiving overload	−0.758	0.168	−4.522	<0.001	−0.265	
Block 3	Intersection	12.317	7.097	1.735	0.084		0.113^**^
	Age	−0.138	0.058	−2.396	0.017	−0.130	
	Type of caregiver	2.706	0.817	3.312	0.001	0.174	
	Role meaning	0.089	0.075	1.175	0.241	0.066	
	Caregiving overload	−0.243	0.190	−1.283	0.201	−0.085	
	Stress management	0.671	0.226	2.973	0.003	0.183	
	Self-confidence	0.533	0.293	1.818	0.070	0.101	
	Work-life balance	0.492	0.234	2.102	0.037	0.145	
	Emotional regulation	0.924	0.317	2.913	0.004	0.164	

^**^p < 0.001.

On the one hand, management of caregiving stress, management of work-life balance difficulties, and emotional self-regulation regulation explained QoL with a positive relationship, that is, higher scores on these variables predicted a higher score on QoL. The type of grandparent caregiver also explained QoL with a positive relationship, which meant that being occasional caregivers predicted a higher QoL score. On the other hand, age explained QoL with a negative relationship, that is, a higher age predicted a lower score on QoL.

## 4 Discussion

The present study provides new data on how grandparents’ sociodemographic variables, grandparents’ role perception and grandparents’ emotional competences predict two health indicators (PW and QoL). Specifically, PW was predicted by age, management of caregiving stress, self-confidence in the caregiving role, management of work-life balance difficulties and emotional self-regulation, while QoL was predicted by age, type of grandparent caregiver, management of caregiving stress, management of work-life balance difficulties and emotional self-regulation.

Concerning the relationship between *age* and PW, the results in the literature are varied. Our results are in line with Noriega et al. (2020), who found a negative relationship between age and personal growth, one of the dimensions of PW. In contrast, other authors have found a positive relationship between the frequency of caregiving and the meaning of life in older grandparents (over 80), as well as a negative relationship in younger grandparents (under 60) (Danielsbacka et al., 2019). Other authors, assessing SW, have found less depressive symptomatology in older grandparents (over 60) when they are moderately involved in the care of grandchildren, while they found no significant differences in younger grandparents (between 45 and 59) (Zeng et al., 2020). These differences may be due, firstly, to the different assessment scales used in the studies and, secondly, to the culture background of each study. While some participants also were Spanish (Noriega et al., 2020), other were Chinese (Zeng et al., 2020), and, in the case of Danielsbacka et al. (2019), the study include different European countries, between which there may be differences.

Regarding the relationship between *age* and QoL, different authors exposed negative relationships between supplementary grandparents caregivers’ QoL and their age in line with our study. Younger grandparents scored higher on the physical and psychological dimensions of health-related QoL (Yalcin et al., 2018; Noriega et al., 2022). As for psychosocial QoL, lower scores had been found in control and autonomy dimensions in younger grandparents (Szabó et al., 2019). According to Yalcin et al. (2018), one explanation could be the fewer physical and psychological resources available for caregiving as the person’s age advances.

Secondly, one of the variables related to *grandparents’ role perception* (type of grandparent caregiver) also act as predictors of QoL. Literature agrees that grandchild caregiving can have negative consequences for grandparents if it becomes too intense. Glaser et al. (2014) exposed that moderate grandchild caregiving was related to higher psychosocial QoL for grandparents. Yalcin et al. (2018) found higher levels of health-related QoL in occasional supplementary caregivers versus primary caregivers. Our results are congruent with these findings, as being an occasional caregiver has been shown to positively predict QoL.

In last place, the role of *emotional competences* in grandparents’ PW is unknown. However, as have been mentioned, different intervention studies have focused on teaching supplementary grandparent caregivers’ psychological resources to improve their health (Kirby and Sanders, 2014; Zauszniewski et al., 2014). Our results are consistent with this, as all the emotional competences, which are similar to the strategies taught in those intervention programs, predicted positively PW. The same was true for QoL, except in the case of self-confidence in the caregiving role.

Regarding *emotional self-regulation*, authors have found relationships between emotional intelligence (EI) and wellbeing in both middle and older adults. Whitmoyer et al. (2024) found positive relationships between two emotional strategies (positive reappraisal and situation selection) and SW (balance between pleasant and unpleasant emotions). Delhom et al. (2017) found significant relationships between EI (attention to feelings, emotional clarity, and repair and regulation of the emotions) and PW dimensions. Concretely, self-acceptance, personal growth and purpose in life were related to the three dimensions of EI (attention, clarity, and repair); autonomy and environmental mastery were only related to clarity and repair; and positive relations with others were not related to any of them. As for QoL, positive relationships between EI and the psychological dimension of health-related QoL were found in middle and older adults (Wilson and Saklofske, 2018). Concretely, Quattropani et al. (2022) found a positive relationship between this dimension and suppression abilities, i.e., the ability to reduce or suppress unpleasant emotions.

Studies on the predictive role of the competences *management of caregiving stress* and *management of work-life balance difficulties* on the PW and QoL of supplementary grandparents are unknown. Even though supplementary care of grandchildren is related to a lower psychological and physical impact than, for example, that of custodial grandparents (Triadó et al., 2014), it has been shown that the impact also depends on the intensity of care. In general, the literature agrees that grandparent caregiving has positive consequences for grandparents when it is not intense and can become negative if it is intense (Burn and Szoeke, 2015). Studying supplementary grandparents caregivers, Triadó et al. (2014) evidenced as the main predictor of their subjective health the perceived difficulties in caring for their grandchildren. This research is in line with ours, in which higher scores in the management of caregiver stress and of work-life balance difficulties predicted higher scores in PW and QoL. Given the caregiving situation in which the participants find themselves, having tools to manage the stress associated with caregiving and reconcile the caregiving role with the other roles they play, could favors the levels of PW and QoL.

Studies assessing *grandparents’ self-confidence* are still unknown, nor is there much research on the relationship between self-confidence and wellbeing. A concept close to self-confidence is self-efficacy, that is, the self-perception of one’s ability to perform a task adequately. Self-efficacy is influenced by pleasant and unpleasant emotions, experiences of success and failure, and positive or negative feedback received from the environment (Bandura, 1977). Together with perceived satisfaction, the feeling of self-efficacy makes up the concept of parental competence (Albanese et al., 2019; Milkie and Nomaguchi, 2020). While parental competence refers to the knowledge, skills, and attitudes necessary to adequately perform the parenting role (Masten and Curtis, 2000), parental self-efficacy refers to parents’ confidence in their ability to overcome parenting challenges and promote their children’s development (Coleman and Karraker, 2000). Although studies analyzing the relationship between parental self-efficacy and wellbeing are scarce, some authors have found negative relationships between parental self-efficacy and some indicators of SW, such as depressive symptoms and negative affect (Teti and Gelfand, 1991; Coleman and Karraker, 1998), as well as with postnatal depression (Haslam et al., 2006). Also, it has been negatively associated with parental stress (Ayala-Nunes et al., 2014). Moreover, positive relationships have been found between parental self-efficacy and role satisfaction (Coleman and Karraker, 2000), and positive parenting practices, such as open and trusting communication or being supportive and responsive (Salo et al., 2022). Although there is no literature available on the relationship between grandparents’ self-efficacy and PW, these data seem to agree with our results, as grandparents’ self-confidence was found to be a predictor of PW.

Several limitations of the present investigation should be mentioned, so that they can be considered for future research. Firstly, this study included a non-probability sample. It is likely that a large majority of the grandparents who participated in this study had a satisfactory experience with their role as supplementary grandparents caregivers, as it is difficult for those with more negative experiences to participate in this type of study. Therefore, it is important to keep in mind that the results may be biased. Secondly, the sample included a greater number of occasional versus regular grandparents caregivers. For future research, it would be appropriate to have similar groups, so that the different experiences of each group can be analyzed. Thirdly, this is a cross-sectional study, so we cannot know whether our results will be stable over time. In the future, longitudinal studies should be developed to further explore this issue. Finally, the results are from Spanish grandparents, so caution should be exercised in generalizing these results, considering cultural differences.

Despite these limitations, our study provides useful information on the role of emotional competences and the grandparents’ perception of their role as predictors of grandparents’ PW and QoL. This is a novel study for several reasons. First, it focuses on PW and psychosocial QoL, whereas most of the literature on grandparent supplementary caregivers deals with subjective wellbeing and health-related QoL. Second, it provides insights into the role of grandparents as supplementary caregivers, an increasingly socially relevant issue, whereas research has tended to focus on grandparents as primary caregivers. Third, in contrast to the usual tendency to focus on the negative consequences that caring for grandchildren may have for grandparents, this study emphasizes the competences that grandparents may have to cope with their role as caregivers. Fifth and finally, related to the above, knowing which emotional competences predict grandparents caregivers’ PW and QoL is key to guiding the development of psychoeducational intervention programs to help them cope adequately with their caregiving role.

In relation to the last mentioned aspect, Spain is characterized by strong family values and, at the same time, few family support policies, which favors the expectation that grandparents help their children in the care of their grandchildren, as a way of compensating for the shortcomings of the system (Gauthier, 1996; Hank and Buber, 2009). Consequently, it would be appropriate to develop psychoeducational programs that include both grandparents and parents, offering them tools to face this reality in a satisfactory way. Taking into account this sociocultural context, it would be appropriate to work on: (1) the non-interference rule, so that grandparents understand what their role as auxiliary caregivers is, respecting the upbringing chosen by the parents; (2) grandparents’ rights, so that they are able to establish their limits and be able to continue developing the rest of their vital areas; (3) communication strategies, to facilitate the adjustment of expectations between grandparents and parents; (4) grandchild care skills, given the large percentage of Spanish grandparents who regularly care for their grandchildren; and finally (5) it would be interesting that future programs include information and tools for grandparents to be updated in new technologies, as this is one of the main generation gaps between grandparents and grandchildren today, being undeniable that the fact that grandparents have these skills can favor closer relationships with their grandchildren.

## Data availability statement

The datasets presented in this study can be found in online repositories. The names of the repository/repositories and accession number(s) can be found at: https://osf.io/unjr4/?view_only=6610a675552b4b59bdbc4e66885143a4.

## Ethics statement

The studies involving humans were approved by Comité de Ética de Investigación de la Universidad San Pablo-CEU. The studies were conducted in accordance with the local legislation and institutional requirements. The participants provided their written informed consent to participate in this study.

## Author contributions

LG: Conceptualization, Formal analysis, Investigation, Methodology, Writing – original draft, Writing – review & editing. CN: Conceptualization, Formal analysis, Funding acquisition, Investigation, Methodology, Supervision, Writing – original draft, Writing – review & editing. GP-R: Conceptualization, Formal analysis, Investigation, Methodology, Writing – original draft, Writing – review & editing. JL: Conceptualization, Formal analysis, Investigation, Methodology, Writing – original draft, Writing – review & editing.
